# Clinical trial: probiotic treatment of acute distal ulcerative colitis with rectally administered *Escherichia coli *Nissle 1917 (EcN)

**DOI:** 10.1186/1472-6882-10-13

**Published:** 2010-04-15

**Authors:** Harald Matthes, Thomas Krummenerl, Manfred Giensch, Corinna Wolff, Jürgen Schulze

**Affiliations:** 1 Department of Gastroenterology, Community Hospital Havelhoehe, Kladower Damm 221, 14089 Berlin, Germany; 2 Private Practice, An der Germania Brauerei 6, 48159 Muenster, Germany; 3 Private Practice, Am Wall 1, 21073 Hamburg, Germany; 4 Ardeypharm GmbH, Loerfeldstr. 20, 58313 Herdecke, Germany

## Abstract

**Background:**

Probiotics are effective in inflammatory bowel diseases. Clinical effectiveness and dose dependency of E. coli Nissle (EcN) enemas were investigated in ulcerative colitis (UC).

**Methods:**

In a double-blind study, 90 patients with moderate distal activity in UC were randomly assigned to treatment with either 40, 20, or 10 ml enemas (N = 24, 23, 23) containing 10E8 EcN/ml or placebo (N = 20). The study medication was taken once daily for at least 2 weeks. After 2, 4 and/or 8 weeks the clinical DAI was assessed together with tolerance to treatment. Patients who reached clinical DAI ≤ 2 within that time were regarded as responders.

**Results:**

According to ITT analysis the number of responders was not significantly higher in the EcN group than in the placebo group (p = 0.4430, 2-sided). However, the Jonckheere-Terpstra rank correlation for dose-dependent efficacy indicated a significant correlation of per-protocol responder rates (p = 0.0446, 2-sided). Time to remission was shortest with EcN 40 ml, followed by EcN 20 ml. The number of adverse events did not differ notably.

**Conclusion:**

In contrast to ITT analysis, efficacy of rectal EcN application was significant in PP and points to EcN as a well tolerated treatment alternative in moderate distal UC.

**Trial registration:**

German Clinical Trials Register DRK00000234.

## Background

Ulcerative colitis (UC) is an inflammatory bowel disease characterised by acute inflammation of the colonic mucosa and intermittent, symptom-free periods of remission [1]. Its aetiology is currently not well understood, but a number of hypotheses including genetic factors, alimentation, autoimmunogenicity, and an imbalanced gut microflora have been put forward [2,3]. Moreover, a number of different disease activity indices have been suggested for use in clinical trials [4]. Although spontaneous remissions have been observed, the disease usually requires treatment. The number of treatment options for distal UC have increased in recent years, essentially by two different approaches: containment of the immune response and modulation of the intestinal microflora [1]. Examples of the former include topical compounds like 5-aminosalicylic acid (mesalazine), steroids, cytoprotective agents, lidocaine, short-chain fatty acids, cyclosporine [1], and enemas [5]. However, prolonged use of chemicals such as corticosteroids may result in serious complications, hence a number of treatment alternatives and various routes of administration are being investigated [6].

Modulation of the intestinal microflora is based on Alfred Nissle's observation [7] that the application of *Escherichia coli *Nissle 1917 (EcN) can have a positive effect on UC. His probiotic hypothesis led to a number of studies. Successful oral application of microorganisms in UC remission induction or maintenance, such as EcN [8], *S. boulardii *[9], bifidobacteria [10], and others [11,12] as well as the role of probiotic functional foods or probiotic therapies in inflammatory bowel disease was reviewed by [13-16]. Furthermore, the effect of probiotics on acute disease was investigated [17], with EcN currently appearing to be one of the most promising candidates. The rational of probiotic treatment was recently further supported by findings of reduced diversity in the dominant faecal microbiota of UC patients [18]. In addition, good tolerance of rectally applied EcN was already been shown in a phase-I study involving 80 patients (data on file at Ardeypharm). In the present trial we investigated the treatment of patients suffering from active UC proctitis/proctosigmoiditis, with 40, 20, or 10 ml EcN enemas (10^8 ^EcN/ml) for 4 to 8 weeks and compared it to placebo.

## Methods

This was an explorative, randomised, double-blind, placebo-controlled, parallel-group, multicentre, phase-II dose-finding study. It was performed in accordance with the requirements of GCP and the Revised Declaration of Helsinki, approved by the independent ethics committee of the Medical Association Westphalia-Lippe, Muenster, Germany, and registered with the German national regulatory authority (Federal Institute for Drugs and Medical Devices submission number No. 4015691).

### Patients

Eligible participants were recruited in 10 centres (1 university hospital, 4 community hospitals, and 5 community based gastroenterologists) in Germany between Nov 1999 and June 2002. All were treated as out-patients. Prior to admission to the trial, each patient was informed by the investigator about the nature, significance, and possible consequences of the trial and its procedure as well as efficacy and adverse drug effects of the trial medication.

All patients gave their express informed consent in written form.

Due to its explorative character, sample size calculation was not possible in this trial. The objective was hypothesis generation. However, the general design of randomised, explorative, phase-II dose-finding studies was taken into consideration. A total of 90 patients between 18 and 70 years of age were admitted to the study, who had a confirmed diagnosis of acute UC proctitis/proctosigmoiditis with mild to moderate disease activity. Active disease was defined as a Disease Activity Index (DAI) according to Sutherland of 4-9 [19]. Proctitis with inflammation beginning at the anus up to ≤ 15 cm, and proctosigmoiditis with inflammation beginning at the anus up to the end of colon sigmoideum (about 25 - 30 cm from anus) were acceptable for inclusion, if verified by endoscopy and histology. Inclusion criteria further stipulated at least two confirmed prior manifestations of disease.

Exclusion criteria were other causes of acute proctitis or proctosigmoiditis such as infections, medical drugs, radiation, ischaemia of affected intestinal segments, and Crohn's disease. A history of stool incontinence, perianal fistulae, major colonic surgery, colorectal carcinoma, or stenoses too, led to exclusion just as other severe accompanying diseases. Participation in another clinical trial either simultaneously or within 30 days prior to enrolment was forbidden by protocol. Also not permissible was medication such as oral EcN within 4 weeks prior to the study, rectal treatment with steroids or aminosalicylates within 2 weeks before the study, immunosuppressants within 90 days before inclusion, and antibiotics or sulphonamides during the study course. Finally, a lack of cooperation, inadequate contraception, pregnancy or breast feeding, drug or alcohol dependency, neurotic personality, and obesity were reasons for exclusion. Permissible concomitant therapies included loperamide drops to improve retention capacity for enemas, and oral UC maintenance treatment with aminosalicylates or steroids at a constant level for at least two weeks prior to the study. However, any dosage alteration led to exclusion from per-protocol analysis. The decision to exclude a patient was taken by an independent steering committee before unblinding of the random code. It was not considered necessary to actively verify the effectiveness of blinding.

### Study medication

The investigational drug was an enema containing probiotic, non-pathogenic Escherichia coli strain Nissle 1917 (manufactured by Ardeypharm, Herdecke, Germany; 10^8 ^viable microorganisms per ml). Other components were purified water, sodium chloride, potassium chloride, magnesium sulfate, magnesium chloride. As placebo an identical enema preparation devoid of the active substance, was used.

Patients were randomly assigned to one of three EcN groups EcN 40 ml, EcN 20 ml, EcN 10 ml or placebo. The placebo group was pooled from three groups that matched the three different enema volumes used in the EcN groups (6 patients received 10-ml enemas, 7 received 20-ml enemas, and 7 received 40-ml enemas). Therefore, blinding was granted with regard to the use of the active substance or placebo.

Enemas were stored in the fridge and ready to use after 30 minutes at room temperature. I.r application was carried out in the evening and maintained once daily for at least 2 weeks. Viability of the study medication was verified regularly.

### Study design

**Figure 1:**
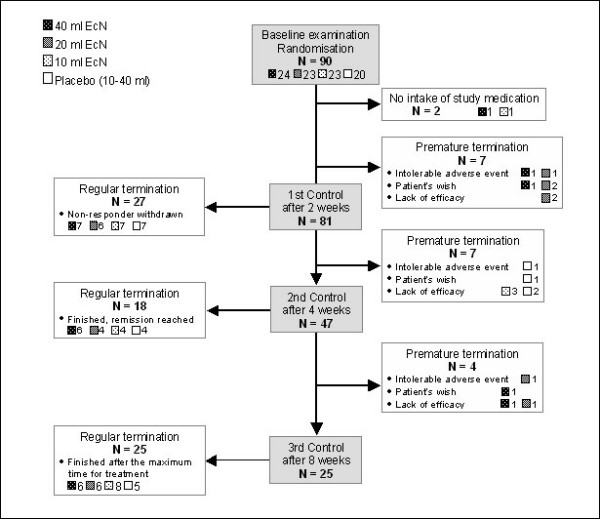
**Patient flow chart**.

Eligible patients were enrolled and randomised to treatment with either 40, 20, or 10 ml EcN (24, 23, and 23 patients, respectively) or placebo (20 patients; Figure 1). Using standard predetermined randomisation tables and the order of enrolment, patients received a randomisation number. No patient was randomised in order to replace a patient who left the study prematurely. Blinding of the investigator and patient was ensured by the provision of study medication identical in appearance, with a patient specific randomisation number.

Patients applied the enemas in the evening before going to sleep and retained it in the rectum for as long as possible. As per protocol, patients left the study after 2, 4, or 8 weeks. If after 2 weeks of treatment health condition had not improved, patients were classified as non-responders and discontinued. This early discontinuation was necessitated by ethical considerations on the use of placebo, even though one can assume that continued treatment would have resulted in higher responder rates. If remission was achieved after 4 or 8 weeks of treatment, patients finished the study and were classified as responders. The flow of patients is shown in Figure 1.

Clinical DAI was defined comprising the parameters "stool frequency", "rectal bleeding" and "assessment of disease activity by physician"; sigmoidoscopies were performed with the initial and final examination. Patients had to keep a diary for daily self-assessments of global health on a 100-mm visual-analogue scale (VAS). An additional scale used for global assessment of health by patients and investigators comprised six categories, i.e. complete recovery, noticeable, moderate or slight improvement, no changes, and deterioration [20]. At control visits (week 2, 4, and 8) these data were combined with additional parameters assessed (e.g. efficacy of the study medication as assessed by patients and investigators, and practicability of treatment as assessed by patients) and transferred to a case report form. Efficacy and practicability of treatment were determined on 5-point scales ("very good", "good", "moderate", "barely satisfactory", "poor"). Empty medication containers were returned to the centres in order to check for patient compliance. At the control visits, safety parameters were also monitored and recorded by the investigators together with all adverse events (AEs).

### Evaluation

The objective of this evaluation was to further characterize the involvement of the gut microflora in ulcerative colitis, hence to test the probiotic hypothesis. The primary outcome measured was the number of patients who reached clinical remission within the study period under EcN therapy versus placebo. Clinical remission was defined as a clinical DAI ≤ 2, which even meets a more recent and stringent classification of patient defined endpoints [21]. Patients with thus defined clinical remission at the last documented control examination (last observation carried forward (LOCF)) were regarded as responders.

Secondary outcome criteria were the time to remission, endoscopic mucosal healing (DAI = 0), and disappearance of histological signs of significant inflammation (according to [22]). In addition, safety monitoring of vital parameters and standard laboratory values were performed. For the assessments of AEs and general tolerance to treatment by the patients and investigators, a 5-point scale again was used (please cf. above).

### Statistical analysis

The one-sided Jonckheere-Terpstra test was used for explorative investigation whether a dose-effect relation existed between the 4 regimens of treatment. The zero hypothesis, equal efficacy of the 4 treatment regimens was tested against the alternative of treatment rankings. This assumes a difference between EcN and placebo and differences amongst the 3 EcN groups with increasing efficacy achieved by larger volumes of EcN. The statistical evaluation for the comparison of the treatments was carried out on the primary target parameter, the remission rate defined as the percentage of patients with clinical DAI ≤ 2.

Fisher's exact test was used to evaluate adverse events. Data on secondary objectives were compared descriptively. Two sets of patients were analysed: An intent-to-treat population (ITT), including all patients who confirmedly took at least one dose of the study medication, and a per-protocol population (PP). Both the ITT and PP evaluations were carried out for all efficacy parameters. Safety and tolerance was analysed using ITT data only. All biostatistical evaluations were performed by ClinResearch GmbH, Cologne, Germany, using the statistical software package SAS^®^, version 8.2.

## Results

### Patient Characteristics

Of 90 patients enrolled in the study, 88 received at least one dose of study medication and provided at least one post-baseline value; hence they were included in the ITT analysis set (Figure 1). 55.7% were male and 44.3% female, all between the age of 21 and 67 years, and a BMI between 16.3 and 46.7 kg/m^2^. There were no apparent differences between the study groups at baseline. Similarly, their case histories were comparable (data not shown). The majority of patients had concomitant medical treatment of their acute UC proctitis/proctosigmoiditis at baseline (Table 1). Mesalazine was the most common antiinflammatory drug while acetylsalicylic acid and paracetamol were primarily used as analgesics. Overall, neither the concomitant diseases nor concomitant therapies varied significantly between the study groups. Abnormalities of colon-descendens histology were observed in 20/88 patients at baseline. These split however evenly between the groups. Before unblinding a steering committee assessed protocol violations in 45/88 patients (51.1%) of the ITT data set (12/23 [52.2%], 9/23 [39.1%], 12/22 [54.5%] in the 40, 20, and 10 ml EcN groups, respectively, as well as 12/20 (60.0%) in the placebo group). Major protocol deviations comprised violations of inclusion criteria, intake of non-permissible concomitant medication for more than 3 days, intake of ≥ 3 g mesalazine or sulfasalazine during the entire study, discontinuation of ≥ 3 g mesalazine immediately before the start of study, intake or application of steroids (≥ 3 mg local or > 10 mg systemic) during the study, and intake of of less than 70% of study medication. Accordingly, the PP analysis set comprised 57 patients (17/23, 18/23, and 11/22 EcN 40, 20, and 10 ml patients, respectively, and 11/20 placebo-treated patients). Premature discontinuation of the study for lack of efficacy occurred in 35/88 patients (7/23, 8/23, and 10/22 in the 40, 20, and 10 ml EcN groups, respectively, and 10/20 in the placebo group). The number of patients in the study at the scheduled visits is shown in Figure 1.

**Table 1:** Demographics and concomitant medication

	Overall	40 ml EcN	20 ml EcN	10 ml EcN	Placebo
**Age (years)**	41.8 ± 12.7	40.5 ± 14.3	42.3 ± 12.0	37.5 ± 9.0	47.4 ± 13.8

**Gender (m : f)**	49 : 39	13 : 10	13 : 10	11 : 11	12 : 8

**Smoking**	3/88	2/23	1/23	0/22	0/20

**Previous colonic surgery**	-	-	2/23	-	1/20

**Antidiarrhoeal, antiinflammatory and/or antiinfective medication**	53/88 60.2%	15/23 65.2%	11/23 47.8%	15/22 68.2%	12/20 60.0%

**Systemic corticosteroids**	14/88 15.9%	3/23 13.0%	3/23 13.0%	6/22 27.3%	2/20 10.0%

### Primary objective

**Figure 2:**
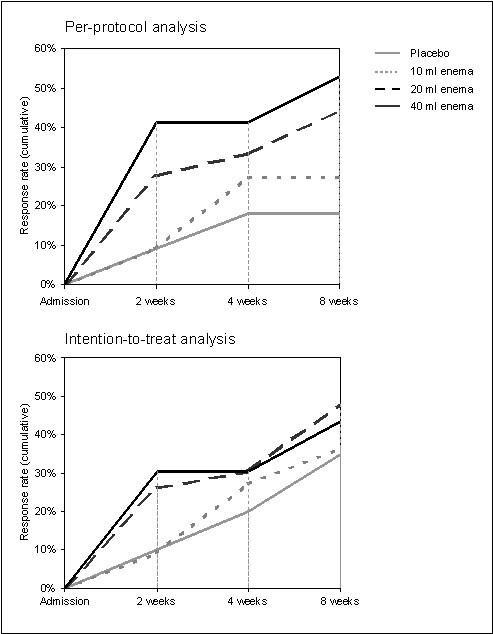
**Time to remission, per-protocol analysis (N = 57) and intention-to-treat analysis (N = 88)**.

Remission rates in 57/90 PP patients were clearly dose-dependent (Figure 2): EcN 40 ml (9/17 [52.9%]), 20 ml (8/18 [44.4%]), 10 ml (3/11 [27.3%]), and placebo (2/11 [18.2%]). As would be expected, this dose-dependency was less pronounced in the ITT analysis (10/23 [43.5%], 11/23 [47.8%], 8/22 [36.4%], and 7/20 [35.0%] in the EcN 40 ml, 20 ml 10 ml, and placebo group, respectively). Jonckheere-Terpstra rank-correlation test for dose-dependent efficacy indicated statistical significance (p = 0.0446 two sided) for PP but not ITT analysis (p = 0.4430 two sided).

### Secondary objectives

**Figure 3:**
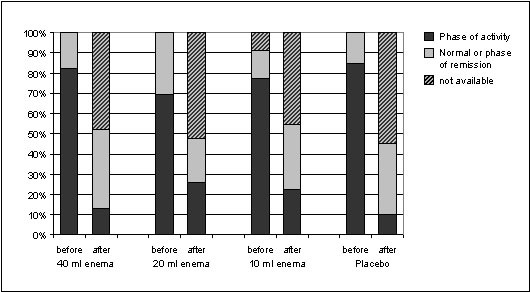
**Changes in histological findings in rectum, intention-to-treat analysis (N = 88)**.

**Figure 4:**
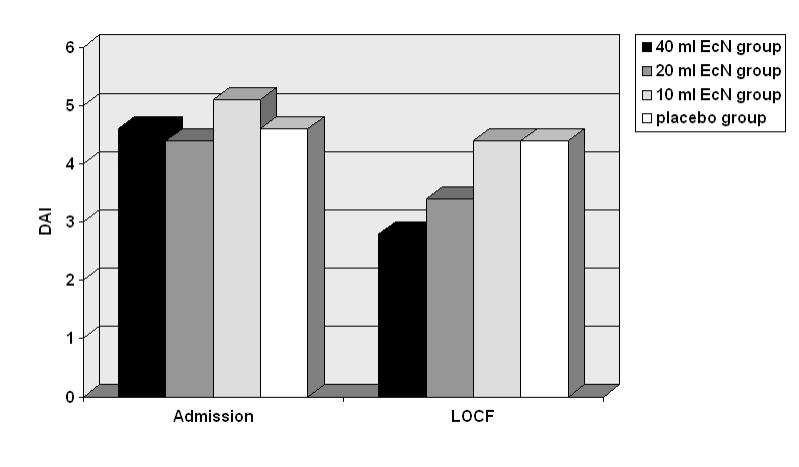
**Course of clinical DAI between admission and end of treatment (LOCF), per- protocol analysis (N = 57)**.

Time to remission was shorter in the 40 ml and 20 ml EcN groups than in the 10 ml EcN and placebo groups (Figure 2). With regard to endoscopy, results were also favourable for EcN. Of all PP patients with abnormal mucosal findings at baseline (N = 57), 8/17 (47.6%, 40 ml EcN), 7/18 (38.9%, 20 ml EcN), 5/11 (45.5%, 10 ml EcN), and 3/11 (27.3%, placebo) showed remission or improvement of the histological score. Similar results were obtained in the ITT population. Vulnerability of the intestinal mucosa and abnormal histological findings in the rectum generally decreased from admission to the last control examination as shown in Figure 3. However, as histology revealed, mucosal healing was most prominent in the EcN 40 ml group. At baseline only 2 PP patients allocated to EcN 40 ml presented remission while 10 patients showed moderate or high-grade disease activity as identified histologically. At the final visit after 8 weeks the number of PP patients in remission had increased to 8, 2 PP patients still showed moderate or high-grade disease activity. A positive development was also seen in the clinical DAI between baseline and LOCF (Figure 4). The same development was seen in the sigmoidum, but due to the limited spread of proctitis it was less distinct (data not shown). Defaecation frequency, occurrence of rectal bleeding, and general disease activity improved in all treatment groups (data not shown).

Backing up the main result, efficacy of the study medication was assessed as "good" or "very good" in 52.2% (12/23), 47.8% (11/23) 40.9% (9/22), and 40.0% (8/20) of cases for EcN 40 ml, 20 ml, 10 ml, and placebo, respectively by the investigators. Similar results were obtained by patients' assessments (data not shown).

In addition to these clinical parameters, practicability of treatment was assessed by the patients. Practicability of Enema application was rated "good" or "very good" by 13/23 (56.5%), 11/23 (47.8%), 12/22 (54.5%), and 9/20 (45.0%) patients in the EcN 40 ml, 20 ml, 10 ml, and placebo groups, respectively. Ratings on the VAS scale and self-assessments by patients corresponded well with those of investigators and clinical findings, but there was no apparent difference between the treatment groups (data not shown). The same is true for the global assessment of health. According to both patients and investigators, the global state of health improved in all treatment groups from admission to LOCF with no difference between EcN and placebo-treated patients.

### Safety

The majority of patients indicated "good" to "very good" tolerance of study medication at LOCF (19/23 [82.6%], 18/23 [78.3%], 17/22 [77.3%] and 18/20 [90.0%] patients) and in the patient diary (17/23 [73.9%], 17/23 [73.9%], 16/22 [72.7%], and 17/20 [85.0%] patients in the EcN 40 ml, 20 ml, 10 ml, and placebo group, respectively). In addition, vital and laboratory parameters did not show any clinically relevant changes from admission to LOCF. No difference between the treatment groups was established (data not shown).

### Adverse events

A total of 47/88 patients (53.4%) experienced at least one adverse event (AE) during the course of the study, with a similar distribution between the groups (10/23 [43.5%], 15/23 [65.2%], 12/22 [54.5%], and 10/20 [50.0%] for EcN 40 ml, 20 ml, 10 ml, and placebo, respectively). The most frequently observed AEs comprised gastrointestinal and thoracic disorders. Twelve of 88 (13.6%) patients experienced a new concomitant disease. Of these AEs, only one (aggravated proctosigmoiditis) in the EcN 20 ml group was regarded as serious. The majority of AEs were "not related" to the study drug. Only in 2/23 (8.7%), 2/23 (8.7%), 1/22 (4.5%), and 1/20 (5.0%) patients in the EcN 40 ml, 20 ml, 10 ml, and placebo group, respectively, it was considered as "possibly" or "probably related". Of these 6 AEs, most were related to flatulence or other gastrointestinal disorders. Due to AEs the study medication was discontinued in 5/23 (21.7%), 7/23 (30.4%), 4/22 (18.2%), and 3/20 (15.0%) patients in the EcN 40 ml, 20 ml, 10 ml, and placebo group, respectively.

## Discussion

Up to now, no standard therapy for UC proctitis/proctosigmoiditis has been established. Systemic or topical modulation of the immune response, e.g. with mesalazine or sulfasalazine (alone or in combination), is a very common treatment option [23] and may reduce the associated risk of colon cancer in UC [24]. However, a variety of side effects have been reported, especially with long-term use [25,26]. Therefore, an alternative treatment option is required.

After several positive studies with EcN capsules in UC remission maintenance [8,27,28] this is the first trial looking at enemas in active disease. Moreover, it is the first study investigating the efficacy of rectally applied EcN in UC. The capsules were enteric-coated in order to survive the gastric juice and open up not before the terminal ileum is reached. As EcN acts locally in the colon this trial, by using enemas, aimed at helping patients with rectal disease even better by bringing the probiotic closer to the focus of inflammation.

*E. coli *strain Nissle 1917 is one of the best characterized strains used as a probiotic drug. Different strain-specific characteristics have been described, e.g. six iron-acquisition systems, secretion of two microcins, formation of biofilms under various conditions, a unique structure of the lipopolysaccharide, and survival in the gut [18,29,30]. Especially with regard to UC, recent research allows for deeper insight into its modes of action. Here, the induction of human beta defensin-2 in enterocytes seems to play a key role in preventing acute attacks [31] with EcN flagellin being an important contributing factor [32].

Following a phase-I study that showed good tolerance in 80 patients (data on file at Ardeypharm), we investigated the efficacy of Mutaflor enemas in a phase-II dose-finding study with clinical remission as the primary endpoint. A clinical DAI ≤ 2 was chosen after clinical observations suggested DAI ≤ 2.5 being equivalent to a "patient-defined remission" [21]. Improvement of endoscopic and histological scores were considered secondary endpoints. This focus on purely clinical aspects was recently vindicated by findings from Higgins and colleagues [33].

A key objective of the treatment of active disease is the reduction of clinical symptoms relevant to the patient. Our data suggest that rectal EcN is probably an effective treatment option. This is corroborated by ratings of the investigators and patients. Enemas with 40 ml seem most effective while still being practical. However, the statistically significant dose response for clinical remission as well as the improved time to remission should be verified by a phase-III study. Especially the high number of patients excluded from the trial for major protocol violations (in particular for intake of non-permissible concomitant medication) necessitates a careful interpretation of the results. Our observation was unexpected since compliance had been good in the previous outpatient-based study. Recruitment through community-based gastroenterologists might have been one contributing factor, but lack of efficacy, especially in the placebo group, could have also encouraged the use of additional medication not allowed by the protocol.

Although continuation of oral remission-maintenance therapy with aminosalicylates or steroids at constant dose may be considered a weakness of this trial, it was the only practicable way to investigate patients with acute UC proctitis and proctosigmoiditis. Disease exacerbations due to discontinuation of oral medication would probably have led to even more drop-outs and raised major ethical concerns.

## Conclusion

A dose-dependent efficacy of rectal EcN compared to placebo was observed in PP patients with active, mild to moderate distal UC while this effect was not shown in the ITT population. EcN may represent an effective and well tolerated alternative or supplementary treatment option to topically applied aminosalicylates or glucocorticoids. However, a confirmatory study in a larger subset of patients is needed in order to gain further evidence. Enemas with 40 ml EcN seem to be most promising.

## Competing interests

The authors declare that they have no competing interests.

## Authors' contributions

All authors conceived, and participated in the design and coordination of the study. HM drafted the present manuscript. All authors read and approved the final manuscript.

## Pre-publication history

The pre-publication history for this paper can be accessed here:

http://www.biomedcentral.com/1472-6882/10/13/prepub
